# A population-based retrospective cohort study of 4,103 pneumoconiosis cases in Zhenjiang, China, 1965–2025: a survival analysis

**DOI:** 10.3389/fpubh.2026.1762998

**Published:** 2026-01-29

**Authors:** Qian Haiyang, Li Yanping, Chen Yan, Xie Shi, Wu Jiayan, Ni Jinfeng

**Affiliations:** 1 Zhenjiang Center for Disease Control and Prevention, Zhenjiang, Jiangsu Province, China; 2 Jiangsu Helin Cement Co., Ltd., Zhenjiang, Jiangsu Province, China

**Keywords:** Cox regression analysis, dust-exposure, occupational disease, pneumoconiosis, survival analysis

## Abstract

**Objectives:**

To identify key determinants of survival outcomes in pneumoconiosis patients, quantify their associations with mortality risk, and develop evidence-based intervention strategies for improving quality of life in this population, grounded in epidemiological findings.

**Methods:**

Life-table analysis was employed to calculate survival probabilities and mortality density during the observation period. Kaplan–Meier methods were used to generate survival curves with intergroup comparisons performed by log-rank tests. Cox proportional hazards regression models were applied to evaluate the independent effects of covariates on survival time.

**Results:**

A total of 4,103 cases had several species of pneumoconiosis, including silicosis, coal worker pneumoconiosis, welder pneumoconiosis, and other pneumoconiosis that accounted for 65.56, 28.22, 1.54 and 4.68% of total pneumoconiosis, respectively. 77.82% of cases were initially diagnosed at Stage I, 18.94% at Stage II, and 3.24% at Stage III. 85.86% of patients remained stable condition, 14.14% upgraded. The overall average survival time was determined as 24.35 ± 9.91 years, the life expectancy reached 37.063 years in total, and the total mortality of patients suffering from pneumoconiosis was 28.03%. With disease progression, mean dust exposure duration, mean survival time, and life expectancy demonstrated progressive declines, whereas the number of people with lower economic levels, the proportion of small and medium-sized enterprises, along with age at onset and mortality rates, exhibited significant increases. Industries (mining, manufacturing), enterprise size (medium scale), first diagnostic stage (Stage III), and earlier age of onset were found as important risk factors for the survival of patients suffering from pneumoconiosis.

**Conclusion:**

Stage III at initial diagnosis was an independent risk factor for mortality, while employment in mining/manufacturing, work in medium-sized enterprises, and younger age at onset also significantly increased the risk of death. Occupational health interventions should therefore focus on high-risk industries and enterprises, and enhance monitoring and management of both early-onset and advanced-stage patients. Delaying disease progression and implementing early health management are of critical public health importance for improving overall survival.

## 1 Introduction

Pneumoconiosis is a chronic disease attributed to the long-term inhalation and deposition of inorganic mineral dust in the lungs during occupational activities, with serious harm and a large number of patients (1). The main pathological manifestations of pneumoconiosis include chronic pulmonary inflammation, persistent pulmonary fibrosis and other substantial irreversible changes that may occur after exposure cessation (2, 3). In 2017, silicosis was the most common type of pneumoconiosis worldwide, followed by coal worker pneumoconiosis (CWP) (4). In addition, known pneumoconiosis also include welder’s pneumoconiosis, graphite disease, carbon black pneumoconiosis and others (5). Pneumoconiosis is a chronic occupational disease that, due to its irreversible pathological changes, is difficult to recover from once contracted. At present, there is no effective treatment for pneumoconiosis, and prevention has always been the main means to reduce the incidence rate and mortality of pneumoconiosis, and causing significant economic burden and health losses to the country and patients.

In China, pneumoconiosis is the leading occupational diseases that need to pay more focus. China suffered from the world’s largest health loss from pneumoconiosis in 2019, accounting for two-thirds of the global health loss from pneumoconiosis (6). In 2023, a total of 12,087 new cases of various occupational diseases were reported nationwide, including 8,051 cases of occupational pneumoconiosis, accounting for 66.61% of the total cases (7). According to occupational history and the severity of radiographs, pneumoconiosis is divided into three stages in China (8), which is different from the classification of simple type (further divided into subtypes 1, 2, and 3) and complex type (further divided into subtypes A, B, and C) by the International Labor Organization (ILO) (9). However, there is a certain correlation between the two. The first, both using lung changes on radiographs as the main basis for diagnostic classification; the second, Stage I represents simple pneumoconiosis of the ILO, while Stage III represents complex pneumoconiosis of categories B and C of the ILO; the third, Stage II falls between the two stages mentioned above; the fourth, both can serve as important basis for occupational injury compensation. In addition, the classification of pneumoconiosis can be used as an important factor for the survival time of cases (3).

The industrial structure of Zhenjiang City, which is dominated by sectors such as metal and non-metal mining, metallurgy, coal mining, non-ferrous metal smelting and processing, high-end equipment manufacturing, new materials, automotive and parts, as well as shipbuilding and marine engineering, stands in sharp contrast to the export-oriented processing model centered on light industry and electronics in Southern China (10, 11), the foundational industrial model focused on energy and heavy chemicals in Northern China (12), the equipment manufacturing model supported by steel and automobiles in Central China (13), and the resource development model emphasizing mining and metallurgy in Southwest China (14). The industrial characteristics of Eastern China, including Zhenjiang, are marked by a combination of diversification and density (15). The region is home to numerous small and medium-sized manufacturing enterprises, leading to a more complex array of occupational hazards. Although no occupational disease incidents had occurred in the past, there are quite a few pneumoconiosis patients. This distinct context endows research on the survival status of pneumoconiosis in this area with unique representativeness and necessity.

To prevent pneumoconiosis, the ILO and the World Health Organization (WHO) have jointly launched a global plan to eliminate pneumoconiosis, with the goal of eradicating pneumoconiosis worldwide by 2030. China has invested a lot of energy and funds in the prevention and control of pneumoconiosis, and has included occupational health in the Healthy China 2030 Plan, and included in the Five-Year Plan for National Economic and Social Development and the State Council’s Safety Production Assessment. The aim of this study is to evaluate the key factors affecting the survival of pneumoconiosis patients in Zhenjiang City, reduce related complications, prolong survival, and provide evidence for improving patients’ quality of life. Therefore, follow-up was conducted on occupational pneumoconiosis patients reported in Zhenjiang City from 1965 to 2025, and the survival status of pneumoconiosis patients was analyzed, aiming to provide scientific basis for the prevention and treatment of pneumoconiosis.

## 2 Methods

### 2.1 Study subjects

**Figure 1:**
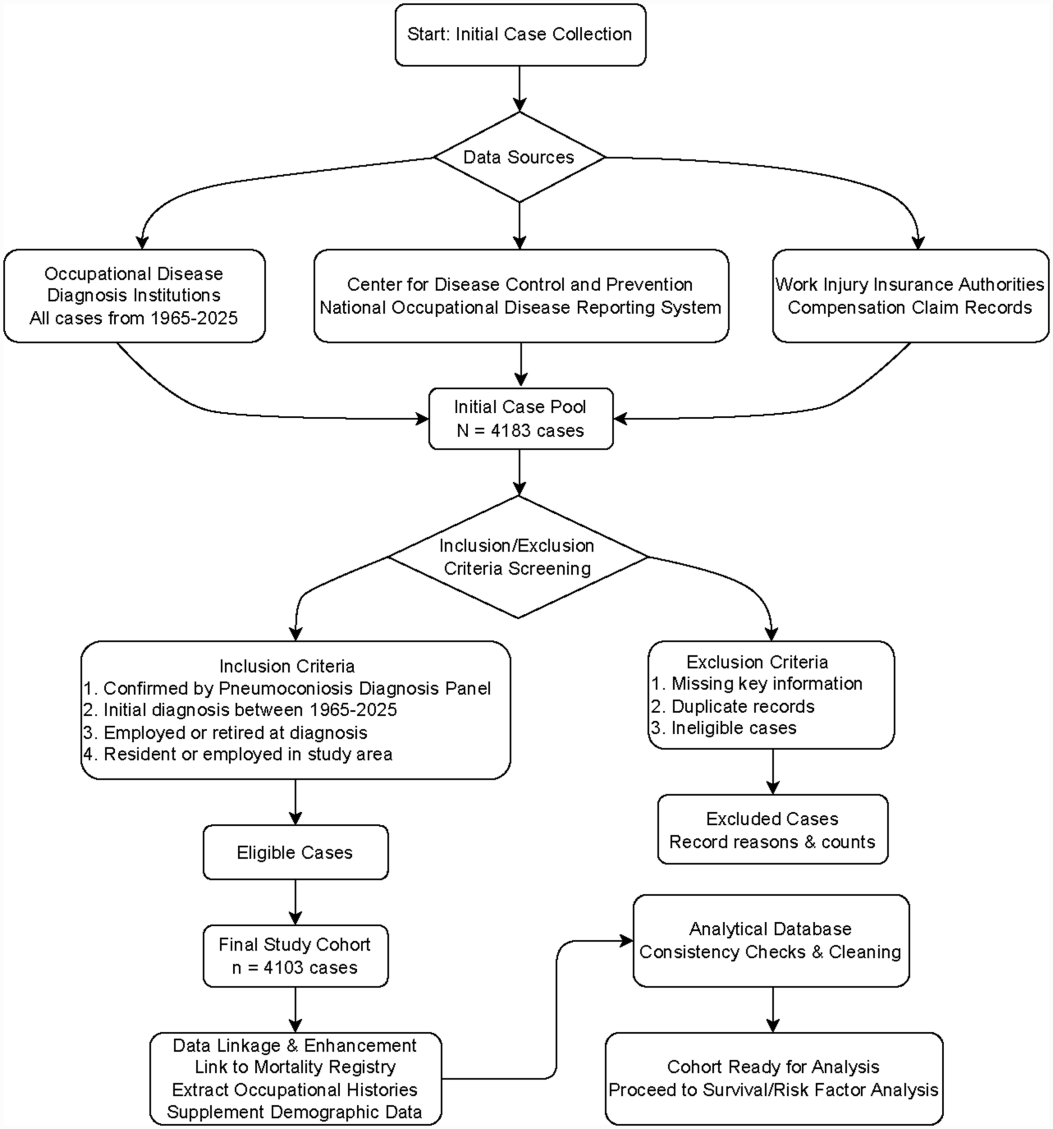
Flowchart of pneumoconiosis case selection and cohort establishment in the Zhenjiang study.

In 2025, a total of nearly 4,200 cases of pneumoconiosis were collected through follow-up and retrospective investigation in Zhenjiang City. Excluding factors such as no onset time, inability to investigate survival status, no exact date of death for death cases, and loss of contact, a total of 4,103 relatively complete data points were screened. Figure 1 shows the flowchart of pneumoconiosis case selection and cohort establishment in the Zhenjiang study. The study population consisted of 4,103 cases of pneumoconiosis first diagnosed in Zhenjiang City from 1965 to 2025. The patients were diagnosed with pneumoconiosis by the Zhenjiang City Center for Disease Control and Prevention, a qualified institution for occupational disease diagnosis. The diagnosis was made by at least three certified occupational disease physicians in accordance with the prevailing “Diagnostic Criteria for Pneumoconiosis.” Due to the continuous revision of diagnostic standards, from 1965 to 2025, the “Medical Preventive Measures for Silicon Dust Workers,” “GB 5906–1986 X-ray Diagnosis Standards and Treatment Principles for Pneumoconiosis,” “GB5906-1997 X-ray Diagnosis for Pneumoconiosis,” “GBZ 70–2002 Diagnosis Standards for Pneumoconiosis,” “GB-70-2009 Diagnosis Standards for Pneumoconiosis” and “GBZ 70–2015 Diagnosis Standards for Pneumoconiosis” were successively adopted for the diagnosis of pneumoconiosis. According to the above criteria, pneumoconiosis was divided into three stages based on the progression of the disease or the severity of the condition. This study was conducted in accordance with the Helsinki Declaration (revised in 2024). This study had been approved by the Ethics Committee of the Zhenjiang Center for Disease Control and Prevention (No. 2025002), and individual consent for this retrospective analysis had been waived.

### 2.2 Data source

The information of pneumoconiosis patients mainly came from three channels: core diagnosis and case registration from occupational disease diagnosis institutions and Centers for Disease Control and Prevention (CDC), occupational exposure history information from employers’ occupational health surveillance archives, work-related injury insurance and labor security records, survival outcomes and cause of death information from the all-cause-of-death registration system, registered residence and population registration system. Data from all the above sources were securely linked and merged using the resident ID number as the unique identifier. In cases of discrepancies between sources (e.g., differences in recorded diagnosis dates), the research team adjudicated based on the original diagnosis certificate or the earliest reliable record to ensure the accuracy and consistency of each variable. All the information of pneumoconiosis patients was obtained and submitted by diagnostic institutions through the occupational disease network direct reporting system. In addition, the occupational disease prevention and control department of the Center for Disease Control and Prevention conducted annual follow-up on pneumoconiosis cases to ensure timely updated of their information. The obtained data involved basic demographic information, job type, industry category, company economic level, initial date of dust exposure, end date of dust exposure, date of first diagnosis of pneumoconiosis, type of pneumoconiosis, diagnosis of each stage, diagnosis date of upgraded outcome variables for each diagnosis stage, date of death, etc. The missing data had check to ensure its completeness and reliability. According to the latest follow-up information in Zhenjiang City, data on 4,103 pneumoconiosis cases from 1965 to 2025 were collected.

### 2.3 Definition

Death cases: Cases of death due to pneumoconiosis or its complications (such as tuberculosis and pulmonary heart disease) were defined as death cases. Using ICD-10 codes, the underlying causes of death for pneumoconiosis cases were primarily categorized under the following codes: J60–J65 (pneumoconiosis due to specific dusts), C45 (mesothelioma attributable to asbestos exposure), J43 (emphysema), C34 (malignant neoplasms of bronchus and lung), A15–A19 (tuberculosis), and I26–I28 (pulmonary heart disease and diseases of the pulmonary circulation).

Censored Cases: Those who died due to other reasons (such as accidents, liver failure, kidney and autoimmune diseases), or were still alive at the end of observation (October 31, 2025), or loss to follow-up, were classified as censored data.

Survival time: It was defined as the period in years from the initial pneumoconiosis diagnosis (t₀) to death (the event), study termination, or loss to follow-up (censoring).

Disability rating: The disability level was determined in accordance with “GB/T16180-2014 Standard for identify work ability—Gradation of disability caused by work-related injuries and occupational diseases.”

### 2.4 Statistical analysis

All statistical analyses, including descriptive statistics and survival analyses, were performed using R software (version 4.5.1). Group comparisons utilized the χ^2^ test for categorical variables and one-way ANOVA for continuous variables. Survival rates and median survival time were estimated via life-table analysis for age-stratified cases. Age-specific mortality probabilities were also derived from these analyses. Mortality rates were compared across subgroups created by the intersection of age categories and disease stages. Kaplan–Meier survival curves were generated and compared using the log-rank test. Independent predictors of survival were identified using a multivariate Cox proportional hazards model. Covariates for the final model were selected through a forward stepwise procedure, guided by the likelihood ratio test and the Akaike Information Criterion (AIC). A two-sided *p*-value < 0.05 was considered statistically significant. Definitions and coding for all variables included in the regression models are provided in Table 1.

**Table 1:** Cox and Weibull regression variable assignment table.

Variable name	Assignment description
Age (X1)	Continuous variable
Gender (X2)	Men = 1, women = 2
Industry category (X3)	Mining = 0, manufacturing = 1, public management and social security = 2, others = 3
Economic level (X4)	High level = 1, medium level = 2, low level = 3
Enterprise size (X5)	Big = 1, middle = 2, small or micro = 3, others = 4
Dust exposure duration (X6)	Continuous variable
Types (X7)	Silicosis = 0, CWP = 1, Welder’s pneumoconiosis = 2, others = 3
First diagnostic stage (X8)	Stage I = 0, stage II = 1, stage III = 2
Age of onset (X9)	Continuous variable
Upgrade of stages (X10)	Stable stages = 0, upgrade I to II = 1, upgrade II to III = 2, upgrade I to III = 3
Social security (X11)	Deny = 0, employment injury insurance = 1, medical insurance = 2
Degree of disability (X12)	Unrated = 0, ≤Level 7 = 1, Level 7–4 = 2, >Level 4 = 3

## 3 Results

### 3.1 Demographic and occupational characteristics

Silicosis (2,690 cases, 65.56%), CWP (1,158 cases, 28.22%), and welder’s pneumoconiosis (63 cases, 1.54%) were the top three of the total reported pneumoconiosis. According to the stage of pneumoconiosis, demographic and occupational characteristics data were divided into three groups. Among all 4,103 participants, 3,193 (77.82%) were first diagnosed in Stage I, 777 (18.94%) in Stage II, and 133 (3.24%) in Stage III. Most patients (3,523 cases, 85.86%) were still in the initial diagnosis stage and remained stable, with 520 cases (12.67%) upgraded from Stage I to Stage II, 37 cases (0.90%) upgraded from Stage II to Stage III, and 23 cases (0.56%) upgraded from Stage I to Stage III. The average ages of the three stages were 72.34 ± 8.20 years, 73.28 ± 9.02 years, and 70.47 ± 12.08 years, respectively. The onset age of Stage III was higher than that of Stage I and Stage II. Contrary to the above results, the average dust exposure period in the Stage III was shorter than that of Stage I and Stage II, and the dust exposure duration was generally between 1 and 52 years. The overall average survival time of all participants was 24.35 ± 9.71 years, with a survival time of 20.55 ± 13.50 years for Stage III, significantly lower than Stage I (23.59 ± 9.10 years) and Stage II (28.13 ± 10.38 years). The overall mortality rate and mortality rates of patients with Stage I, Stage II, and Stage III pneumoconiosis were 28.03, 24.12, 38.10, and 63.16%, respectively. The mortality rate showed an upward trend as the pneumoconiosis stage progressed. All variables in the three stages had statistical significance (*p* < 0.05; Table 2).

**Table 2:** Demographic and occupational characteristics involved in this study.

Variables	Stage I (*n* = 3,193)		Stage II (*n* = 777)		Stage III (*n* = 133)		F	*p*
	N	%	N	%	N	%		
Age (years), mean ± SD	72.34 ± 8.20		73.28 ± 9.02		70.47 ± 12.08		7.60	<0.001
<50	25	0.78	12	1.54	7	5.26		
50–60	275	8.61	57	7.34	19	14.29		
60–70	646	20.23	146	18.79	32	24.06		
≥70	2,247	70.37	562	72.33	75	56.39		
Gender							10.35	<0.001
Male	3,151	98.68	773	99.49	128	96.24		
Female	42	1.32	4	0.51	5	3.76		
Industry category							82.43	<0.001
Mining	2,284	71.53	672	86.48	88	66.17		
Manufacturing	575	18.01	57	7.34	26	19.55		
Public management and social security	318	9.87	47	6.05	19	14.29		
Others	19	0.60	1	0.13	0	0.00		
Economic level							75.31	<0.001
High level	2,484	77.80	678	87.26	77	57.89		
Medium level	398	12.46	61	7.85	26	19.55		
Low level	311	9.74	38	4.89	30	22.56		
Enterprise size							158.56	<0.001
Big	2,352	73.66	665	85.59	74	55.64		
Middle	407	12.75	34	4.38	12	9.02		
Small or micro	115	3.60	31	3.99	27	20.30		
Others	319	9.99	47	6.05	20	15.04		
Dust exposure duration (years), mean ± SD	17.53 ± 7.66		17.14 ± 5.98		15.49 ± 7.37		5.46	0.004
<10	622	19.48	101	13.00	35	26.32		
10–20	1,428	44.72	482	62.03	73	54.89		
20–30	999	31.29	183	23.55	19	14.29		
30–40	128	4.01	7	0.90	6	4.51		
≥40	16	0.50	4	0.51	0	0.00		
Types							102.36	<0.001
Silicosis	1,968	61.63	615	79.15	107	80.45		
CWP	1,000	31.32	140	18.02	18	13.53		
Welder’s pneumoconiosis	53	1.66	8	1.03	2	1.50		
Others	172	5.39	14	1.80	6	4.51		
First diagnostic stage							3300.00	<0.001
Stage I	3,193	100.00	520	66.92	50	37.59		
Stage II	0	0.00	257	33.08	10	7.52		
Stage III	0	0.00	0	0.00	73	54.89		
Age of onset (years), mean ± SD	48.74 ± 9.23		45.15 ± 8.79		49.92 ± 12.62		49.43	<0.001
10–20	3	0.09	1	0.13	1	0.75		
20–30	38	1.19	15	1.93	3	2.26		
30–40	360	11.27	181	23.29	28	21.05		
≥40	2,792	87.44	580	74.65	101	75.94		
Upgrade of stages of pneumoconiosis							4400.00	<0.001
Stable stages	3,193	100.00	257	33.08	73	54.89		
Upgrade I to II	0	0.00	520	66.92	0	0.00		
Upgrade II to III	0	0.00	0	0.00	37	27.82		
Upgrade I to III	0	0.00	0	0.00	23	17.29		
Social security							162.95	<0.001
Deny	771	24.15	297	38.22	85	63.91		
Employment injury insurance	1,533	48.01	258	33.20	27	20.30		
Medical insurance	889	27.84	222	28.57	21	15.79		
Degree of disability							2000.00	<0.001
Unrated	752	23.55	279	35.91	79	59.40		
≤Level 7	2,242	70.22	46	5.92	3	2.26		
Level 7–4	197	6.17	441	56.76	31	23.31		
>Level 4	2	0.06	11	1.42	20	15.04		
Survival time (years), mean ± SD	23.59 ± 9.10		28.13 ± 10.38		20.55 ± 13.50		81.93	<0.001
Outcome variables							144.64	<0.001
Dead	770	24.12	296	38.10	84	63.16		
Survival	2,423	75.88	481	61.90	49	36.84		

SD, standard deviation.

### 3.2 Mean survival time of patients suffering from pneumoconiosis in different age groups

Table 3 presents the cumulative survival rates and mean survival times for pneumoconiosis patients stratified into 10 5-year age groups. The cumulative survival rate declined significantly with increasing age (χ^2^ = 650.18, *p* < 0.001). Notably, patients aged 55–60 years and those over 70 years exhibited longer mean survival times compared with other age groups. The corresponding numbers of death cases in each age stratum are also provided in the table.

**Table 3:** Cumulative survival rate and its median survival time of 4,103 patients suffering from pneumoconiosis.

Age groups	Observed patients	Surviving patients	Observed died patients	Cumulatively observed patients	Corrected observed patients	Mortality rate	Survival rate	Cumulatively survival rate	Cumulatively mortality rate	Hazard rate	Median survival time (years)
<35	2	0	2	4,103	4,103	0.0005	0.9995	0.9995	0.0005	0.6670	0.26
35-	4	0	4	4,101	4,101	0.0010	0.9990	0.9985	0.0015	0.4000	5.03
40-	9	2	7	4,097	4,096	0.0017	0.9983	0.9968	0.0032	0.1670	9.04
45-	29	9	20	4,088	4,083.5	0.0049	0.9951	0.9920	0.0080	0.0581	7.99
50-	109	53	56	4,059	4,032.5	0.0139	0.9861	0.9783	0.0217	0.0064	15.18
55-	242	159	83	3,950	3,870.5	0.0214	0.9786	0.9581	0.0419	0.0046	37.59
60-	356	237	119	3,708	3,589.5	0.0332	0.9668	0.9291	0.0709	0.0038	29.82
65-	468	292	176	3,352	3,206	0.0549	0.9451	0.8862	0.1138	0.0063	30.22
70-	1,336	1,098	238	2,884	2,335	0.1019	0.8981	0.8282	0.1718	0.0014	38.46
≥75	1,548	1,103	445	1,548	996.5	0.4466	0.5534	0.7197	0.2803	0.0013	41.92

### 3.3 Age-specific mortalities for pneumoconiosis cases at the respective stage

Table 4 lists the age-specific mortality rates and median survival time for each stage. In the three stages of the older adult group, there were a considerable number of observed cases and deceased patients. Compared with Stage I, Stage II and Stage III have higher mortality rates and shorter median survival times. Statistical analysis revealed significant disparities in mortality rates between Stage I and Stage II (*p* < 0.05). Additionally, a statistically significant difference in median survival time was observed between Stage I and Stage III (*p* < 0.05).

**Table 4:** Age-specific mortalities for patients with pneumoconiosis in each stage.

Age	Stage I				Stage II				Stage III			
	Observed patients	Died patients	Mortality rate (%)	Median survival time (years)	Observed patients	Died patients	Mortality rate (%)	Median survival time (years)	Observed patients	Died patients	Mortality rate (%)	Median survival time (years)
<35	0	0	-	0.00	0	0	-	0.00	2	2	100.00	0.24
35-	2	2	100.00	0.05	2	2	100.00	4.33	0	0	-	0.00
40-	6	4	66.67	1.91	2	2	100.00	9.04	1	1	100.00	0.00
45-	17	11	64.71	8.07	8	5	62.50	7.99	4	4	100.00	2.19
50-	78	35	44.87	18.00	19	13	68.42	13.32	12	8	66.67	14.37
55-	197	60	30.46	24.28^a^	38	18	47.37	16.53	7	5	71.43	22.11
60-	289	82	28.37	35.19	53	29	54.72	24.19	14	8	57.14	23.49
65-	357	121	33.89	31.11	93	44	47.31	31.25	18	11	61.11	24.36
70-	1,082	172	15.90	38.46	229	55	24.02	38.05	25	11	44.00	36.11
≥75	1,165	283	24.29	41.95	333	128	38.44	42.97	50	34	68.00	33.86

a Average survival time. Because over 50% of patients were still alive at the end of the study, the median survival time was not reached.

### 3.4 Comparison of survival curves between the three-stage groups

**Figure 2:**
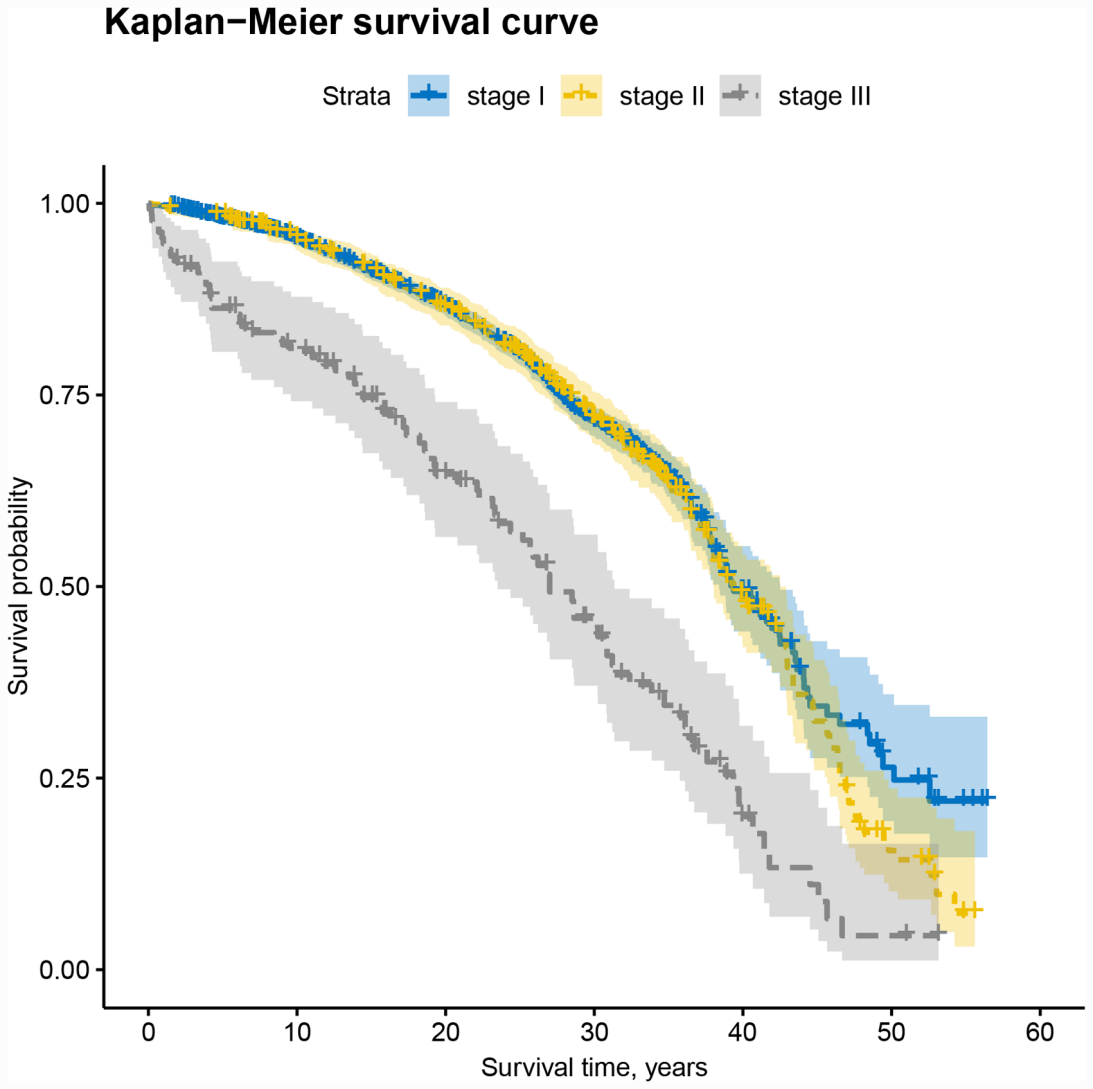
Survival curves of 4,103 pneumoconiosis patients in three stages.

Figure 2 demonstrates distinct survival patterns across pneumoconiosis stages. The survival curves for Stage I and Stage II patients consistently positioned above those of Stage III patients. Specifically, at the 40-year post-diagnosis timepoint, Stage I patients maintained significantly higher survival rates compared to Stage II, although their survival trajectories largely overlapped during the preceding period. Survival analysis revealed statistically significant differences in survival time across stages (*p* < 0.001). All stages exhibited progressively declining cumulative survival rates over time.

The overall life expectancy of the cohort reached 37.063 years (95% CI: 36.2–38.1). When stratified by stage, life expectancy estimates were 38.344 years (95% CI: 37.3–39.4) for Stage I, 37.083 years (95% CI: 35.9–38.3) for Stage II, and 26.441 years (95% CI: 24.8–28.1) for Stage III. Kaplan–Meier analysis with log-rank testing yielded a chi-square value of 66.722, confirming statistically significant differences in survival distributions among the three stages (*p* < 0.001).

### 3.5 Multivariate cox regression analysis of pneumoconiosis cases

Survival analysis was conducted by a Cox regression model. The covariates comprised both categorical variables (e.g., gender, industries category, economic level, enterprise size, disease types, first diagnostic stage, upgrade of stages of pneumoconiosis, and degree of disability) and continuous variables (dust exposure years and age at onset). As shown in the Cox regression analysis results in Table 5, industries (mining, manufacturing), enterprise size (medium scale), first diagnostic stage (Stage III), and age of onset were associated with mortality from pneumoconiosis. The manufacturing industry [Hazard Ratio (HR) = 9.856, 95% confidence interval (CI): 1.724–56.338], mining industry (HR = 2.676, 95% CI: 2.050–3.493), and medium-sized enterprises (HR = 2.493, 95% CI: 1.478–4.204) were reported as the top three risk factors, followed by first diagnosis of Stage III (HR = 1.781, 95% CI = 1.281–2.476) and age of onset (HR = 1.097, 95% CI: 1.088–1.105). The presence of a disability rating—particularly at level 7 or below, or within levels 7–4—was associated with a markedly reduced mortality risk (HR ≈ 0.060).

**Table 5:** Multivariate Cox regression analysis of 4,103 patients suffering from pneumoconiosis.

Variables	β	Standard error	Wald χ^2^	*p*	HR	95% CI
Gender			6.920	0.009		
Male					1.000	
Female	−1.076	0.409	−2.631	0.009	0.341	0.153–0.760
Industries category			19.620	<0.001		
Mining	0.984	0.136	7.236	<0.001	2.676	2.050–3.493
Manufacturing	2.288	0.889	2.572	0.010	9.856	1.724–56.338
Public management and social security	0.453	0.594	0.764	0.445	1.574	0.491–5.038
Others					1.000	
Economic level			7.229	<0.001		
High level					1.000	
Medium level	−1.559	0.428	−3.640	<0.001	0.210	0.091–0.487
Low level	−0.032	0.217	−0.147	0.883	0.969	0.633–1.483
Enterprise size			6.338	<0.001		
Big	−0.096	0.186	−0.513	0.608	0.909	0.631–1.309
Middle	0.914	0.267	3.426	<0.001	2.493	1.478–4.204
Small and micro	0.086	1.003	0.086	0.932	1.090	0.153–7.789
Others					1.000	
Dust exposure duration (years)	−0.004	0.005	0.754	0.385	0.996	0.986–1.006
Types			0.394	0.757		
Silicosis	−0.038	0.075	−0.506	0.613	0.963	0.831–1.115
Coal worker’s pneumoconiosis	−0.186	0.420	−0.444	0.657	0.830	0.364–1.891
Welder’s pneumoconiosis	−0.246	0.256	−0.961	0.336	0.782	0.474–1.291
Others					1.000	
First diagnostic stage			6.004	0.003		
Stage I					1.000	
Stage II	−0.005	0.105	−0.044	0.965	0.995	0.810–1.223
Stage III	0.577	0.168	3.431	<0.001	1.781	1.281–2.476
Age of onset	0.092	0.004	551.883	<0.001	1.097	1.088–1.105
Upgrade of stages of pneumoconiosis			3.486	0.015		
Stable stages					1.000	
Upgrade I to II	−0.255	0.089	−2.871	0.004	0.775	0.651–0.922
Upgrade II to III	−0.218	0.193	−1.126	0.260	0.804	0.551–1.175
Upgrade I to III	−0.401	0.273	−1.471	0.141	0.669	0.392–1.143
Degree of disability			396.038	<0.001		
Unrated					1.000	
≤Disability level 7	−2.880	0.096	−30.124	<0.001	0.056	0.047–0.068
Disability level 7 ~ 4	−2.757	0.143	−19.277	<0.001	0.064	0.048–0.084
>Disability level 4	−2.850	0.721	−3.954	<0.001	0.058	0.014–0.238

## 4 Discussion

Pneumoconiosis is still a threat to public health (16–20). From the global perspective, in 2017, pneumoconiosis had the prevalent cases of 527,500 and over 60,000 new reported cases (21), and its prevalence had increased since 1990 from 0.0054 to 0.0069% (22). An updated report showed, pneumoconiosis was estimated to account for 0.9 million collectively DALYs (Disability-Adjusted Life Years) and 3.1 million prevalent cases in 2019. Pneumoconiosis prevalence has remained comparable from 1990 to 2019 while the ASR (age-standardized rates) of DALYs, deaths, and incidence have decreased by 44.4, 53.3, and 13.7%, respectively. Although the overall trend of the above indicators is declining, the ASR of incidence slightly rose by 5.4% from 1990 to 2019 in women globally, and men have had significantly higher ASR of DALYs, deaths, prevalence, and incidence throughout the investigated period (23). According to recent studies, the number of global pneumoconiosis cases increased by approximately 66.0% from 1990 to 2017, and by 61.5% from 1990 to 2019 (23, 24), and the mortality rate of pneumoconiosis patients has been at a high level in recent years, with over 21,000 deaths each year since 2015 (2), and the rate of pneumoconiosis mortality decreased by 41% from 1990 to 2016 (17).

Different countries or regions still face severe challenges in the prevention and treatment of pneumoconiosis. Pneumoconiosis has re-emerged in the United States and Australia, countries with highly developed healthcare systems, high standards of workplace safety procedures, and highly mechanized mining practices that reduce workers’ exposure to particles (2, 20). In 2018, a study reported that the national prevalence of pneumoconiosis in long-tenured working miners exceeded 10% (25, 26). During the same period, National Institute for Occupational Safety and Health (NIOSH) data showed a sharp increase trend in the incidence of coal worker’s pneumoconiosis (CWP) and progressive massive fibrosis (PMF) (26, 27), and the estimated prevalence of coal workers pneumoconiosis in the 2000s exceeded that in the 1990s (28). Other underdeveloped countries, especially those with inadequate reporting systems, may have many patients who have not yet been diagnosed and reported.

Some reports showed that the age distribution was concentrated in the 60–69 age group, with a higher number of deaths and a greater disease burden. And, the other relevant indicators of older workers were higher than those of younger workers, indicating that the disease burden of employees increases with the increase of dust exposure years (29). In males, the mortality rate of pneumoconiosis increases with age, and the disease burden of middle-aged and older adult people is significantly higher than that of young people (30). The extension of working years leads to the accumulation of dust exposure, increased lung tissue damage, and increased risk of disease (31), with age effects having a greater impact on silicosis and coal worker pneumoconiosis (32).

A survival analysis was conducted in a cohort of 4,103 pneumoconiosis patients diagnosed in an industrial city of China. The study encompassed major pneumoconiosis types, including silicosis, coal worker’s pneumoconiosis, welder’s pneumoconiosis, and other variants (e.g., asbestosis, cement pneumoconiosis, foundry worker’s pneumoconiosis, and potter’s pneumoconiosis). The study was predominantly male, a demographic pattern consistent with established epidemiological characteristics reported in multiple previous studies (1, 13, 24). This demographic distribution reflects significant gender-based occupational segregation within the labor market, wherein high-risk, dust-exposed occupations are predominantly held by males. Survival analysis revealed that patients diagnosed with Stage III pneumoconiosis exhibited a significantly shorter mean survival time and life expectancy compared to those in Stages I and II. Furthermore, Cox proportional hazards regression identified several factors significantly associated with increased mortality risk among pneumoconiosis cases: employment in the manufacturing sector, medium-sized mining enterprises, diagnosis at Stage III at initial presentation, and older age at onset. Of these, the manufacturing industry was associated with the highest hazard ratio, followed by medium-sized mining enterprises, initial diagnosis at Stage III, and older age at onset.

An analysis of the distribution of pneumoconiosis cases in the region identified silicosis, coal worker’s pneumoconiosis, cement pneumoconiosis, and welder’s pneumoconiosis as the four most prevalent types. This disease profile differs notably from the patterns reported in Guangdong and Hunan provinces (33), consistent with Liaoning Province (34), reflecting distinct local industrial structures and associated occupational dust exposures. By disease stage, pneumoconiosis cases were predominantly diagnosed at Stage I (77.8%), with Stage II and Stage III comprising the remaining cases. This stage distribution pattern is consistent with epidemiological findings reported from other regions of China, including Shanghai, Hubei, and Tianjin (35–39). The high proportion of cases diagnosed at Stage I suggests that occupational health surveillance policies, including regular physical examinations, may have played a critical role in early detection. Significant differences were observed in the mean age of onset and dust exposure duration across different disease stages. These variations are associated with several exposure-related and host factors, including cumulative dust exposure time, dust concentration, free silica content, individual susceptibility, and timeliness of medical examination and diagnosis among pneumoconiosis cases (40).

Furthermore, even after cessation of dust exposure, pneumoconiosis demonstrates persistent progression, characterized by further deterioration of lung pathology and progressive decline in pulmonary function, leading to escalation in disease stage. Compared with other types of pneumoconiosis, silicosis progresses faster and more severely (41). Therefore, the diagnosis of stage II or III pneumoconiosis was not only associated with the timing of initial diagnosis but also reflects disease progression from earlier stages. The analysis in this study identified that cases diagnosed at Stage II or Stage III were predominantly concentrated in small and medium-sized enterprises (SMEs) within the mining and manufacturing sectors, which were associated with lower regional economic levels. The contributing factors can be summarized as follows: first, poor working conditions, lack of protective engineering controls, and inadequate implementation of occupational health management systems in these enterprises; second, a general lack of occupational safety training among dust-exposed workers, resulting in insufficient awareness of the health risks posed by dust, particularly silica; and finally, failure to effectively implement relevant occupational health supervision measures, including non-compliance with regular workplace dust monitoring and periodic occupational health examinations for at-risk workers (1). Thus, a significantly shorter dust exposure duration was observed among cases diagnosed at Stage II, and particularly at Stage III, when compared to Stage I cases. This pattern suggests a more aggressive disease progression in these patients.

Silicosis is widely recognized as the most prevalent, rapidly progressive, and clinically severe form of pneumoconiosis (42, 43). This study confirms that silicosis (65.56% of all cases) constitutes the principal component of the pneumoconiosis disease burden. Stratified by disease stage, silicosis accounted for 61.63% (1,968/3,193) of Stage I cases, 79.15% (615/777) of Stage II, and 80.45% (107/133) of Stage III. This study revealed significantly shorter median survival time in Stage III compared to Stages I and II, a pattern potentially explained by the substantially higher proportion of silicosis cases in Stage III. Disease progression analysis demonstrated shorter time to progression to Stage II and Stage III among silicosis patients compared to other pneumoconiosis types. Specifically, 66.92% (520/777) of Stage II cases progressed from Stage I, while 56.07% (60/133) of Stage III cases evolved from earlier stages. The overall and stage-specific mortality rates in this study substantially exceeded those reported in multiple previous studies (3, 44). The high proportion of silicosis across all disease stages may represent a key epidemiological factor underlying this discrepancy, further supporting evidence that silicosis carries higher mortality than other pneumoconiosis types (32). Notably, diagnosis at Stage III was associated with shorter dust exposure duration (1, 45). Despite their relatively brief exposure history, a substantial proportion of these patients were directly classified as Stage III at initial diagnosis. Given the highest proportion of silicosis cases was observed in Stage III, this subgroup may represent distinct exposure patterns or disease progression characteristics compared to other stages.

Analysis of social security coverage among pneumoconiosis patients in this study revealed statistically significant differences across disease stages (*p* < 0.05). While most of Stage I and Stage II patients were enrolled in at least one type of social security scheme—either occupational injury insurance or basic medical insurance—the coverage dropped markedly in Stage III patients. Less than 40% of Stage III patients had dual insurance coverage, and over 60% lacked any form of social security. Among Stage I, Stage II and Stage III patients, this coverage gap was consistent with findings reported by Li et al. (46). Notably, 27% of all patients had not undergone disability assessment, a figure that rose to nearly 60% among Stage III patients. This directly disqualifies a significant number of patients, particularly those with advanced disease, from receiving statutory occupational injury benefits. Existing evidence suggests that comprehensive social security can facilitate timely medical service utilization by improving relevant support mechanisms (4). Furthermore, Wang et al. identified poor economic status as a key barrier to healthcare-seeking among pneumoconiosis patients (47). From a public health perspective, these findings highlight the need for targeted policy interventions: first, to expand social security coverage, particularly for occupational groups at high risk of disease progression; second, to optimize a systematic disability assessment system ensuring patients receive entitled benefits in a timely manner; and finally, strengthen the employer’s main responsibility in occupational disease prevention through multi departmental cooperation.

This study revealed a mean survival time among all patients, which demonstrated significant associations with both dust exposure duration and age at first diagnosis. The combination of shorter dust exposure duration and older age at diagnosis contributed to reduced survival time, further highlighting the long-term health hazards associated with occupational dust exposure. From the perspective of disease natural history, pneumoconiosis typically exhibits a prolonged latency period ranging from 10 to 40 years (6). The powerful compensatory capacity of the pulmonary system, coupled with the non-specific nature of early clinical manifestations, often leads to insufficient healthcare-seeking awareness among patients and consequent delays in diagnosis, thereby creating challenges for early detection and intervention (32). Consequently, most cases are diagnosed at an advanced age and have already progressed to relatively severe stages. Furthermore, the generally declined immune function in older adult patients increases their susceptibility to comorbidities such as tuberculosis and chronic obstructive pulmonary disease (48). Such comorbid conditions significantly elevate both the complexity of clinical management and mortality risk (49). Therefore, it is necessary to conduct timely intervention and treatment for patients with pneumoconiosis to delay the disease progression and prevent potential infections and complications (4).

Life-table analysis of the 4,103 pneumoconiosis patients demonstrated an increasing overall mortality probability and median survival time with advancing age. This positive association between age and mortality probability has been consistently documented in numerous survival analyses. Some studies on occupational-attributable deaths and DALYs from pneumoconiosis indicated that age-standardized mortality rates were highest in older age groups, with the peak DALY rate observed in the 75–84 year age group (17, 41), and another study showed that mortality and DALYs rates peak between the ages of 85 and 89 (29). The observed increase in median survival time may be attributed to the predominance of older age groups within the observed patients, which often represent relatively healthier survivors, thereby contributing to an overall reduction in mortality rates (32), and the risk rate had decreased. Existing research has shown that patients with Stage I pneumoconiosis can receive free standardized rehabilitation treatment after being diagnosed with pneumoconiosis, and their survival time can reach the level of normal individuals (46, 50). The present study involved a substantial proportion of subjects from older age groups, characterized by their advanced age at onset and the fact that approximately 80% of cases were initially diagnosed at Stage I.

Stratified by age and disease stage, mortality demonstrated a progressive increase across the three pneumoconiosis stages, accompanied by corresponding declines in life expectancy. Stage III, characterized by the most severe pulmonary function impairment and a spectrum of comorbidities, exhibited significantly elevated mortality risk compared to Stage I and Stage II. Furthermore, a positive correlation was observed between patient age, disease stage, and comorbidity burden, collectively contributing to reduced survival time (48, 51). The disease burden was substantially higher in middle-aged and older adult populations compared to younger groups (32). As illustrated by the cumulative survival curves, survival probabilities decreased with advancing disease stage. This pattern contrasts with findings reported by Ortmeyer et al., which indicated comparable overall life expectancy between miners and the general population (50). A 20-year follow-up study of South Welsh miners and ex-miners revealed similar survival patterns among subjects with simple pneumoconiosis, category A pneumoconiosis, and those without radiological evidence of the disease (52). Notably, elevated mortality was exclusively documented among miners with category B and C complicated pneumoconiosis. These collective findings underscore that preventing and delaying disease progression represent critical interventions for significantly extending survival time in pneumoconiosis patients (53).

The Cox proportional hazards model identified several independent risk factors for reduced survival in pneumoconiosis patients: employment in the mining sector (HR = 2.676, 95% CI: 2.050–3.493), manufacturing industry (HR = 9.856, 95% CI: 1.724–56.338), medium-sized enterprises (HR = 2.493, 95% CI: 1.478–4.204), diagnosis at Stage III at initial presentation (HR = 1.781, 95% CI: 1.281–2.476), and age at onset (HR = 1.097, 95% CI: 1.088–1.105). The consistent concentration of pneumoconiosis cases in mining and manufacturing sectors, as documented in multiple studies (54–58), reflects the elevated occupational health risks in these industries, including high exposure concentrations to respirable crystalline silica, inadequate engineering controls, and insufficient occupational health surveillance systems. Especially, the patients worked in the mining industry had a worse survival than those worked in other industries (4). Medium-sized enterprises (55, 59, 60), while functioning as crucial contributors to socioeconomic stability through employment generation, exhibit distinct occupational health risk patterns. Epidemiologic analysis revealed that compared to large corporations, these enterprises demonstrated structural disadvantages in allocating resources for occupational health protections. Conversely, when compared with micro-scale economic entities, they encompassed larger workforce populations with broader occupational exposure profiles. This intermediate risk pattern positions medium-sized enterprises as critical observation units in pneumoconiosis disease burden research, carrying significant public health implications for overall working population health. The associations between initial diagnosis at Stage III, age at onset, and patient survival observed in this study align with established risk patterns documented in previous epidemiological study (61). Patients who received a disability rating—especially at level 7 or below, or within levels 7–4—exhibited a significantly reduced mortality risk. This suggests that enrollment in a systematic occupational disease management network may provide patients with more regular medical follow-up and targeted interventions, thereby contributing to improved clinical outcomes.

To date, no survival analysis focusing on pneumoconiosis patients has been conducted in Zhenjiang. This study addresses this gap through a retrospective cohort study designed to systematically examine survival patterns and determinants in this population. Moving beyond descriptive epidemiological characterization, we employed Kaplan–Meier methodology for survival probability estimation and Cox proportional hazards regression to identify independent risk factors. Unlike cross-sectional studies limited to describing disease distribution, survival analysis enables the investigation of factors influencing disease prognosis. Methodologically, this retrospective follow-up study inherits limitations inherent to its design, including potential information bias and loss to follow-up. Furthermore, the absence of precise historical dust exposure metrics, together with the recall bias associated with retrospectively collected behavioral data (e.g., smoking history), constrains the analysis. These gaps highlight the critical importance of designing future studies with prospective, systematic data capture to strengthen the evidence base.

## 5 Conclusion

This study elucidates the key determinants of mortality risk in pneumoconiosis, and its findings offer critical insights into the severe challenges currently facing occupational health in China. The extended latency period of pneumoconiosis and China’s accelerating population aging pose substantial challenges to disease control. This is particularly relevant for the estimated 280 million migrant workers who are predominantly employed in high-risk sectors such as mining, construction, and manufacturing (62). The high mobility of this population contributes to fragmented occupational health supervision systems. Against this backdrop, our findings demonstrate that the risk of death from pneumoconiosis is closely associated with occupational environment (manufacturing, mining, medium-sized enterprises), late-stage detection (Stage III at initial diagnosis), and earlier age at onset. In contrast, the specific etiological type of pneumoconiosis (e.g., silicosis or coal worker’s pneumoconiosis) showed no independent association with mortality. Furthermore, enrollment in the occupational disease disability management system may have a protective effect on patient prognosis.

With ongoing industrial expansion and improvements in diagnostic capabilities, reported pneumoconiosis incidence was projected to rise (25). Therefore, based on these findings, public health interventions should focus on dust control in high-risk workplaces, early diagnostic screening, and prioritized health management for older adult patients and those with advanced disease. Concurrently, it is essential to enhance occupational health surveillance mechanisms and implement differentiated prevention and management strategies tailored to specific industries, populations, and disease stages (63).

We propose a comprehensive public health approach comprising the following evidence-based interventions:

i) Strengthen regulatory enforcement in dust-intensive industries through improved engineering controls, personal protective equipment standardization, and mandatory health monitoring systems to facilitate early disease detection and intervention.
ii) Establish standardized quality control metrics for pneumoconiosis management based on disease-specific characteristics, creating evidence-based protocols for delaying progression and improving quality of life.
iii) Develop specialized rehabilitation networks, such as pneumoconiosis rehabilitation station, providing staged interventions for different pneumoconiosis types, effectively reducing complication risks through periodic therapeutic regimens.
iv) Enhance health literacy among at-risk populations while optimizing allocation of medical resources to establish life-course health management systems covering all occupational groups.
v) This multilayered strategy aims to ultimately reduce the national burden of pneumoconiosis through coordinated prevention, standardized treatment, and comprehensive rehabilitation frameworks.

## Data Availability

The original contributions presented in the study are included in the article/supplementary material, further inquiries can be directed to the corresponding author.
